# Prevalence of *Clostridioides difficile* contamination in healthcare and non-healthcare environments: a global systematic review and meta-analysis

**DOI:** 10.1093/inthealth/ihaf111

**Published:** 2025-10-23

**Authors:** Mohammad Hasan Rabiee, Lida Abdolmohammadi Khiav, Mohammad Hossein Fallah Mehrabadi

**Affiliations:** Department of Epidemiology, Razi Vaccine and Serum Research Institute, Agricultural Research, Education and Extension Organization (AREEO), Karaj 31975-148, Iran; Department of Anaerobic Bacterial Vaccine Production and Research, Clostridia Research Laboratory, Razi Vaccine and Serum Research Institute, Agricultural Research, Education and Extension Organization (AREEO), Karaj 31975-148, Iran; Department of Epidemiology, Razi Vaccine and Serum Research Institute, Agricultural Research, Education and Extension Organization (AREEO), Karaj 31975-148, Iran

**Keywords:** *Clostridium difficile*, environment, healthcare, prevalence, systematic review

## Abstract

*Clostridioides difficile* infection is a significant public health challenge. This study aimed to determine the overall prevalence of *C. difficile* contamination in healthcare and non-healthcare environments around the world from 2014 to 2024. In this systematic review, we searched five international databases. Random and fixed-effects models were used to estimate the pooled prevalence with 95% CIs. Among the 49 eligible studies and 20 779 environmental samples, 4507 samples were positive for *C. difficile* contamination. The overall prevalence was estimated as 0.26 (95% CI 0.23 to 0.28). The pooled prevalence according to the detection method was 0.26 (95% CI 0.21 to 0.32) and 0.62 (95% CI 0.50 to 0.74) using a culture test and PCR, respectively. The overall prevalence was 0.18 (95% CI 0.16 to 0.20) and 0.36 (95% CI 0.30 to 0.42) in the healthcare and non-healthcare environment, respectively. The prevalence in non-healthcare environments was 0.42, 0.38, 0.33 and 0.04 on farms, in public places, in the home and veterinary hospitals, respectively. The findings reveal a significant prevalence of *C. difficile* contamination across various environments worldwide. This alarming trend underscores the pressing need to prioritize environmental factors and implement effective disinfection strategies as part of comprehensive control programs aimed at tackling this disease.

## Introduction

*Clostridium difficile* or Clostridioides *difficile* is an anaerobic Gram-positive bacterium that is found as a flora of healthy infants. This bacterium can cause disease with severe diarrhea and inflammation of the colon or colitis, and overuse of antibiotics facilitate its occurrence.^1,2^ The bacterium produces two exotoxins, toxin A (TcdA) and toxin B (TcdB), which are associated with the severity of the disease. Toxin A (308 kDa) causes inflammation, resulting in the secretion of intestinal fluids and colonic mucosal damage. Toxin B (270 kDa) also causes mucosal damage. Furthermore, *C. difficile* produces CD, including a cell binding component (CDTb) and an enzymatic component (CDTa) with ADP ribosyltransferase activity, leading to cytoskeleton disorganization. Other virulence factors include endospore, S-layers, flagella and fimbriae, as well as other virulence factors.^3^

The occurrence of the disease known as *C. difficile* infection (CDI) is associated with clinical symptoms that include watery diarrhea, abdominal pain, dehydration, fever, nausea and weight loss. In other words, the disease can cause a range of gastrointestinal issues from mild to severe that can be life-threatening.^4^ Considering the importance of the disease, Centers for Disease Control and Prevention (CDC) classified CDI as an urgent public health issue in 2013; there were 250 000 infections and 14 000 deaths reported by the CDC in 2013. The crude incidence rate of CDI was reported as 130.28 per 100 000 persons by the CDC in the CDI Surveillance report in 2017. According to the latest update of the report, the crude incidence rate of CDI in 2022 was 116.1 cases per 100 000 persons.^5^

It has been proven that, besides asymptomatic carriers, the environment plays a crucial role in the transmission of CDI, particularly within healthcare settings. Contaminated surfaces in hospitals can harbor *C. difficile* spores, which are highly resistant to environmental stressors and can persist for months or even years. These spores can be transferred to patients through direct contact with contaminated surfaces or via healthcare workers, contributing to the spread of infection. Enhanced cleaning protocols, including the use of sporicidal disinfectants, are essential to mitigate the risk.^6,7^

To date, several researchers have reported the contamination rate in various healthcare and non-healthcare environmental settings. Despite this, no attempt has been made to summarize the reported contamination rate worldwide in recent years. Therefore, to fill this gap and improve our understanding of the current global prevalence of contamination, this study was conducted to evaluate the overall prevalence of *C. difficile* contamination in both healthcare and non-healthcare environments in the last decade (2014–2024).

## Methods

### Search strategy

We conducted a global systematic review by searching five international databases, namely, PubMed, Science Direct, Springer, CABI Direct and Google Scholar. The search covered the period from January 2014 to January 2024 and utilized the following keywords: ‘*Clostridium* spp.’, ‘*difficile* AND environment’, ‘*difficile* AND healthcare’, ‘*difficile* AND equipment’ and ‘*difficile* AND public’. We searched for these combinations in the titles, keywords and abstracts of published papers in the aforementioned databases. The study protocol was registered in the open science framework (OSF) registries for systematic reviews with the following doi: https://doi.org/10.17605/OSF.IO/G8BTQ.

### Study selection process

Studies with insufficient data, irrelevant and duplicated data were excluded from the analysis. Scientist evaluated studies related to *C. difficile* contamination by screening their titles and abstracts, then reviewed the full text of papers for quality assessment after eliminating duplicates. In addition, the references of eligible papers were searched to find additional eligible articles. Scientist conducted separate assessments of the articles' quality.

### Eligibility criteria

Articles were considered eligible if they met the following inclusion criteria: (i) they were conducted on environmental samples; and (ii) they reported the prevalence of *C. difficile* contamination. However, papers that met the following criteria were excluded from the analysis: (i) they were conducted before 2014; (ii) they did not report the prevalence of *C. difficile* contamination; or (iii) they were case reports or experimental studies.

### Data extraction

The investigators used a form specifically designed to extract data from eligible papers. Data from the papers were extracted into summary tables containing information on author(s) name(s), publication year, detection methods, country, region, setting, sample type, total number of samples and the total number of confirmed *C. difficile* samples.

### Quality assessment

The authors reviewed eligible research articles using Critical Appraisal Skills Programme (CASP) Checklists to evaluate cross-sectional studies. This tool has special items to evaluate the quality of a paper, using 10–12 items for each checklist. These checklists evaluate the strengths and weaknesses of articles, the quality of the study design and the applicability of studies. The precise checklist was applied to each paper according to the type of study. For each item, the possible answers were ‘Yes’, ‘No’ or ‘Can’t tell’. Scientist assessed the articles’ quality separately, and discrepancies were resolved via group discussion. For each item, an answer of ‘Yes’ was scored as 1, while answers of ‘No’ or ‘Can’t tell’ were set as 0. Therefore, the maximum score of cross-sectional studies in this systematic review was 11. Articles with scores of ≥75% of the total score were classified as good quality. Articles with scores of 25–75% of the total score were classified as moderate quality articles and articles with scores of <25% of the total score were classified as poor quality articles.^8^

### Statistical analysis

We employed both random and fixed-effects models to calculate the pooled prevalence and 95% CIs. To evaluate heterogeneity among the studies, we utilized Cochran’s Q test and the I^2^ index. A random-effects model was selected for estimation when the I^2^ index was >50% and the p-value from Cochran’s Q test was <0.1. Forest plots illustrate the proportions of individual studies, the pooled prevalence and the heterogeneity across studies. The meta-analysis was performed using Stata version 14.

## Results

### Search results and eligibility

**Figure 1.**
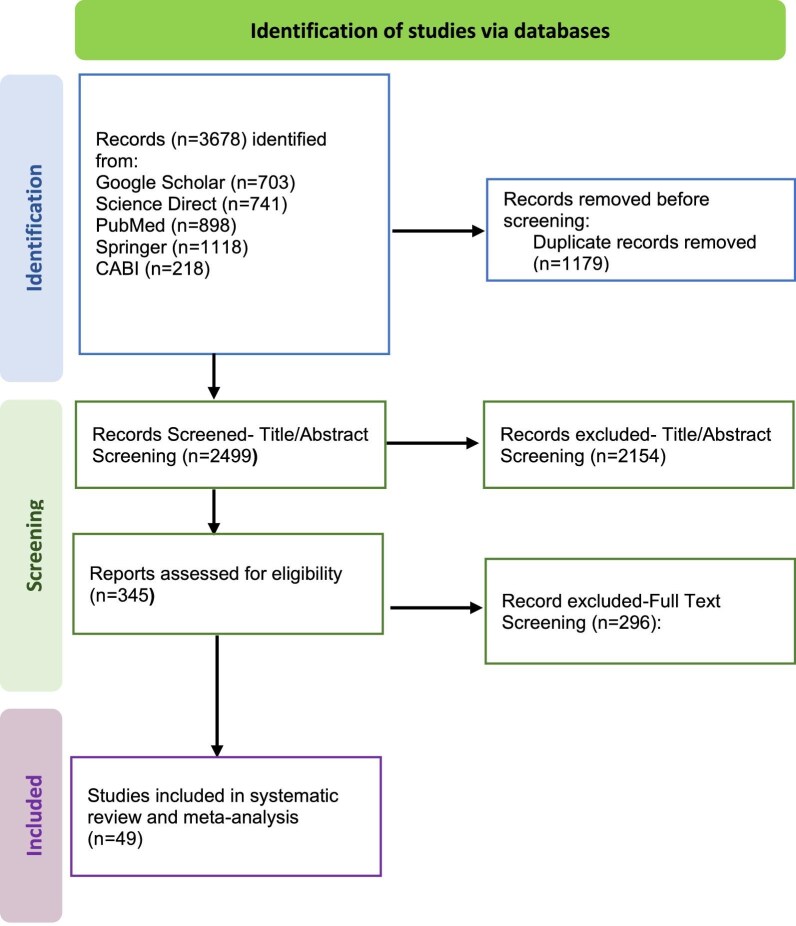
PRISMA flowchart presenting the selection of articles analyzed in this systematic review and meta-analysis. PRISMA: Preferred Reporting Items for Systematic reviews and Meta-Analyses.

Initially, 3678 articles were obtained from the electronic database search. However, 3629 (98.86%) were excluded due to irrelevancy, duplicated documents, lack of access to the full text or insufficient data. Finally, 49 (1.33%) were deemed eligible and subjected to analysis.^6–54^ A flowchart illustrating the screening of papers is presented in Figure 1. Extracted data from studies included in the meta-analysis (up to 1 January 2024) are presented in Table 1.

**Table 1.** Characteristics of the studies included in the meta-analysis

Study number	First author	Year	Country	Setting	Diagnostic methods	Sample size number	Positive number	Reference
1	Aliramezani	2019	Iran	Healthcare	Culture/other test	1435	2	^9^
2	Büchler	2022	Switzerland	Healthcare	Culture/other test	206	37	^10^
3	Doughari	2021	Nigeria	Healthcare	Culture	150	18	^11^
4	Islam	2019	Bangladesh	Non-healthcare	Culture	94	11	^12^
5	Janezic	2018	Slovenia	Non-healthcare	Culture/other test	90	31	^13^
6	Janezic	2020	Slovenia	Non-healthcare	Culture	25	12	^14^
7	Jinhee	2022	USA	Healthcare and non-healthcare	-	6966	1794	^15^
			Mexico, Peru, Guatemala, Brazil, France, Germany, Italy, Taiwan, India	Non-healthcare	-	891	194	
8	Kabała	2022	Poland	Healthcare	Culture/other test	116	6	^16^
9	Kiersnowska	2022	Poland	Healthcare	Culture	116	6	^17^
10	Lemiech-Mirowska	2023	Poland	Healthcare	Culture	100	7	^18^
11	Lim	2020	Australia	Healthcare	Culture	302	159	^19^
12	Perumalsamy	2019	Australia	Healthcare	Culture	159	96	^20^
13	Reigadas	2020	Spain	Healthcare	Culture/other test	476	73	^21^
14	Riley	2018	Australia	Non-healthcare	PCR	145	86	^22^
15	Shivaperumal	2021	Australia	Non-healthcare	Culture	97	65	^23^
16	Rodriguez	2019	Belgium	Non-healthcare	Culture/other test	112	45	^24^
17	Sofjan	2020	Bangladesh	Healthcare	Culture	400	44	^25^
18	Srinivasa	2019	USA	Healthcare	Culture/other test	101	14	^26^
19	Villagomez-Estrada	2019	Spain	Non-healthcare	Culture/other test	100	4	^27^
20	Weppner	2021	USA	Healthcare	Culture	76	17	^28^
21	Zhou	2021	China	Healthcare	Culture	608	12	^29^
22	Blau	2023	Germany	Non-healthcare	Culture and other test	79	30	^30^
23	O’Shaughnessy	2019	Canada	Non-healthcare	Culture/other test	350	144	^31^
24	Simecka	2019	USA	Healthcare	Culture/other test	630	24	^32^
25	Khun	2023	Vietnam	Healthcare and non-healthcare	Culture	199	65	^33^
26	Warren	2022	USA	Healthcare	Culture	758	121	^34^
27	Lim	2022	Australia	Healthcare	Culture	600	185	^35^
28	Endres	2018	USA	Healthcare	Culture/other test	50	14	^36^
29	Tsai	2022	Taiwan	Non-healthcare	Culture/other test	29	7	^37^
30	Moradigaravand	2018	UK	Non-healthcare	Culture/other test	20	18	^38^
31	Garey	2020	USA	Healthcare	Culture	659	55	^39^
32	Shoaei	2019	Iran	Healthcare	Culture/other test	92	9	^40^
33	Odoyo	2020	Kenya	Healthcare	Culture	57	43	^41^
					PCR	57	43	
34	Brown	2018	Canada	Healthcare	PCR	96	50	^42^
					Culture	48	21	
35	Moono	2017	Australia	Non-healthcare	Culture/other test	311	182	^43^
36	Alam	2017	USA	Healthcare and non-healthcare	-	2538	418	^44^
37	Orden	2018	Spain	Non-healthcare	Culture/other test	40	21	^45^

**Table 1.** Continued

Study number	Author	Year	Country	Setting	Diagnostic methods	Sample size number	Positive number	Reference
38	Taha	2022	Egypt	Non-healthcare	Culture	200	38	^46^
39	Alves	2022	Portugal	Non-healthcare	Culture/other test	60	35	^47^
40	Redding	2021	USA	Non-healthcare	Culture/other test	92	28	^48^
41	Usui	2017	Japan	Non-healthcare	Culture/other test	14	5	^49^
42	Marcos	2021	Ireland	Non-healthcare	Culture/other test	180	68	^50^
43	Alam	2014	USA	Non-healthcare	Culture/other test	127	41	^51^
44	Leitner	2020	Austria	Healthcare	Culture	92	0	^52^
45	Ali	2021	India	Non-healthcare	Culture/other test	6	1	^53^
46	Lim	2020	Australia	Non-healthcare	-	142	32	^54^
47	Janezic	2016	Slovenia	Non-healthcare	Culture/other test	183	44	^55^
48	Baghani	2020	Iran	Non-healthcare	Culture/other test	72	1	^56^
49	Shaughnessy	2016	USA	Non-healthcare	Culture/other test	233	31	^57^

### Characteristics of eligible studies

In general, 49 studies measured *C. difficile* contamination in healthcare and non-healthcare settings. Among these, 25 (51.02%) measured contamination in healthcare settings. Non-healthcare settings consisted of farms, public places, homes and veterinary hospitals. Of the 49 eligible studies, 21 (42.85%) were published before 2020. Based on WHO regions, two were conducted in Africa, while 13, 16, four, three and 11 were conducted in America, Europe, Eastern Mediterranean, South-East Asia and Western Pacific, respectively.

### Quality of the eligible studies

Of the 49 original articles, considering the total CASP scores, 31 (63.26%) met all the approval criteria; 46 (93.87%) were classified as good quality and three (6.13%) were classified as moderate quality. No articles were classified as being of poor quality.

### Prevalence of *C. difficile* contamination

**Figure 2.**
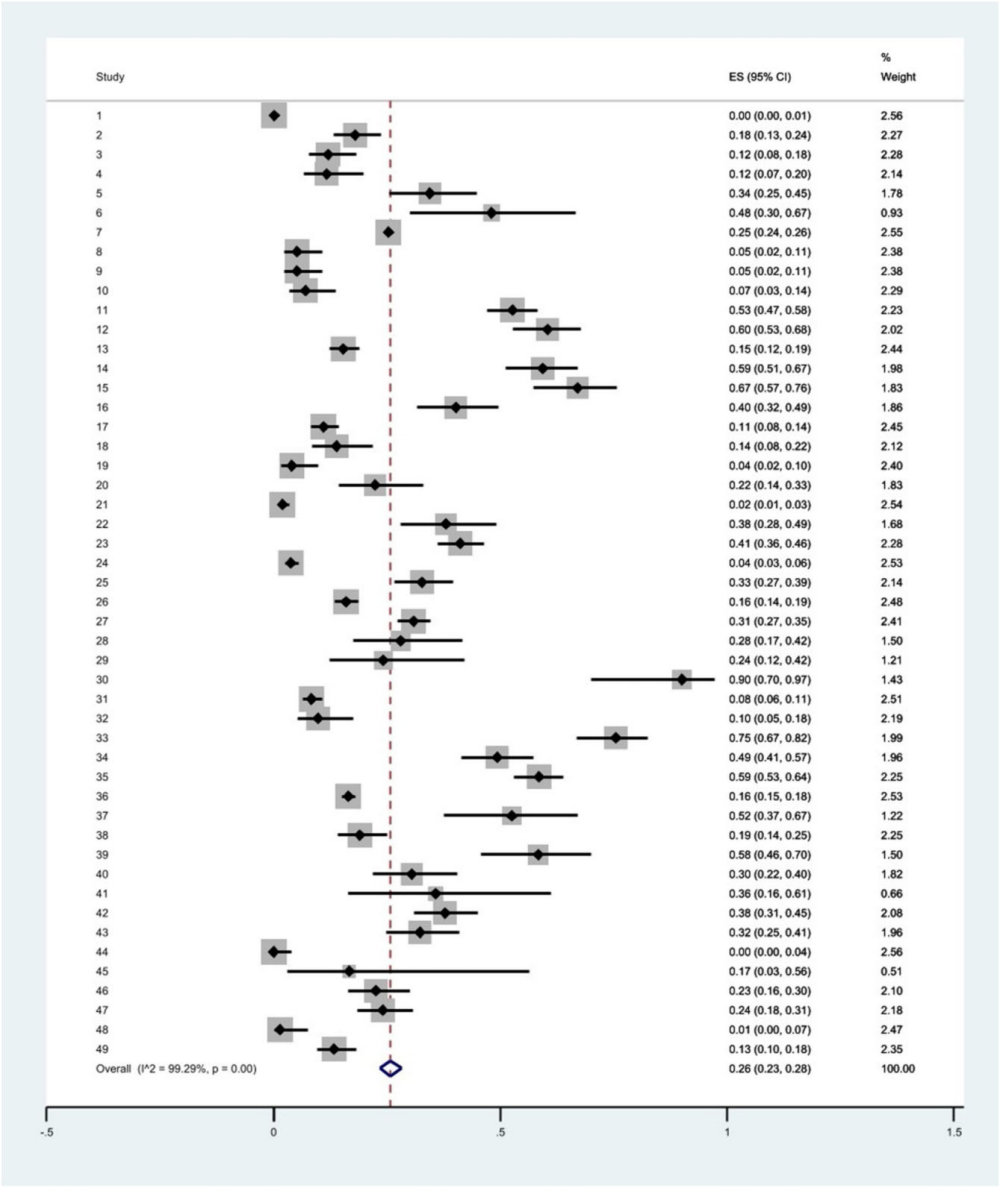
Forest plot of the pooled prevalence of *C. difficile* contamination in healthcare and non-healthcare environments.

Overall, 20 779 environmental samples were investigated in healthcare and non-healthcare settings. Among these, 4507 samples were positive for *C. difficile* contamination using different detection methods. Meta-analysis indicated that the pooled prevalence of *C. difficile* contamination using random effects was estimated as 0.26 (95% CI 0.23 to 0.28). There was a high degree of heterogeneity in the prevalence estimates between different studies. It was observed that the Q statistic was 6767.31 (df=48), p<0.01 and I^2^=99.29% (Table 2 and Figure 2).

**Table 2.** Pooled prevalence of *C. difficile* contamination in healthcare and non-healthcare environments

			Total			Heterogeneity			Heterogeneity
	Number	Sample	Number	Pooled					between
Independent variable	of studies	size	positive	prevalence	95% CI	Q statistic	I^2^ (%)	p	subgroup bias
Diagnostic test									
Culture	19	4740	975	0.26	0.21–0.32	1709.52	98.95	0.00	0.00
Culture and other test	26	5204	915	0.27	0.21–0.33	1655.63	98.49	0.00	
PCR	3	298	179	0.62	0.50–0.74	0	0	0.00	
Region									
Africa	2	264	104	0.31	0.27–0.36	-	-	-	0.00
Eastern Mediterranean	4	1799	50	0.07	0.01–0.13	56.58	94.70	0.00	
America	13	12 724	2772	0.23	0.17–0.29	813.01	98.52	0.00	
South-East Asia	3	500	56	0.11	0.08–0.14	-	-	-	
Western Pacific	11	2606	894	0.41	0.23–0.58	1365.82	99.27	0.00	
Europe	16	1995	437	0.28	0.20–0.36	815.86	98.16	0.00	
Year									
Before 2020	21	6968	1311	0.31	0.25–0.38	2294.83	99.13	0.00	0.11
After 2020	28	13 811	3196	0.24	0.19–0.30	4415.21	99.39	0.00	
Setting									
Healthcare	25	11 475	1962	0.18	0.16–0.20	2929.39	99.18	0.00	0.00
Non-healthcare	30	9304	2545	0.36	0.30–0.41	1157.38	97.49	0.00	
Sample type									
Soil	8	530	310	0.59	0.47–0.71	60.19	88.37	0.00	0.00
Shoe	8	2084	813	0.34	0.23–0.45	156.27	95.52	0.00	

**Figure 3.**
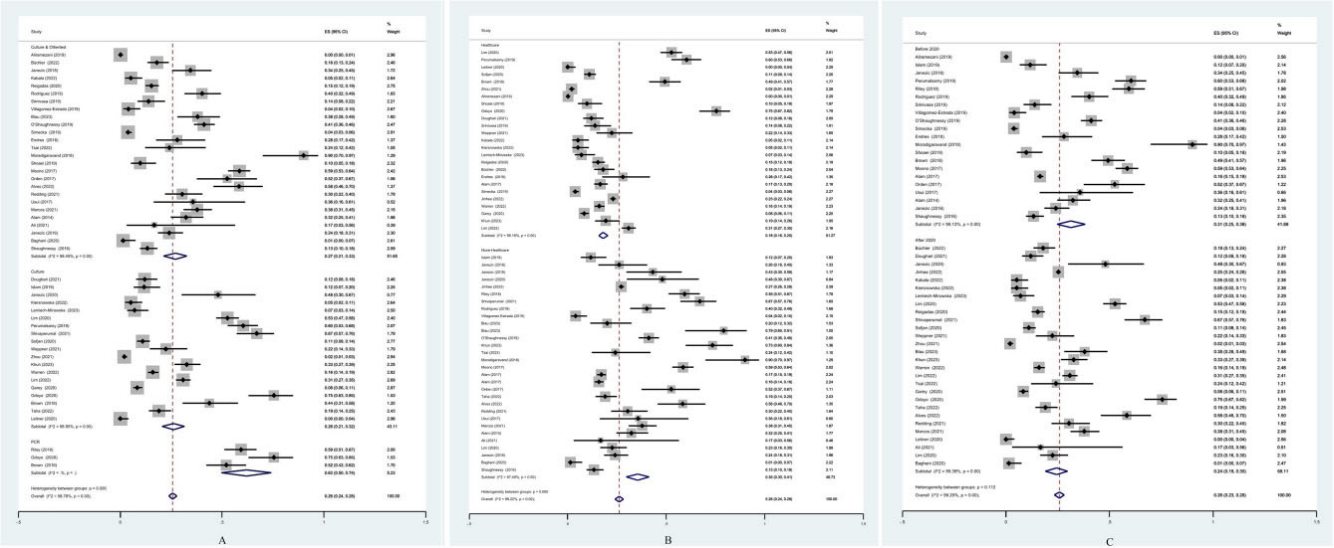
Forest plots of the pooled prevalence of *C. difficile* contamination in healthcare and non-healthcare environments according to (A) diagnostic test, (B) setting and (C) year.

According to diagnostic method, the pooled prevalence of *C. difficile* contamination by culture test was 0.26 (95% CI 0.21 to 0.32). The pooled prevalence using PCR was 0.62 (95% CI 0.50 to 0.74). Meanwhile, the pooled prevalence using a mixed method was 0.27 (95% CI 0.21 to 0.33) (Table 2 and Figure 3).

The pooled prevalence of *C. difficile* contamination was 0.31 (95% CI 0.25 to 0.38) and 0.24 (95% CI 0.19 to 0.30) before and after 2020, respectively (Figure 3).

**Figure 4.**
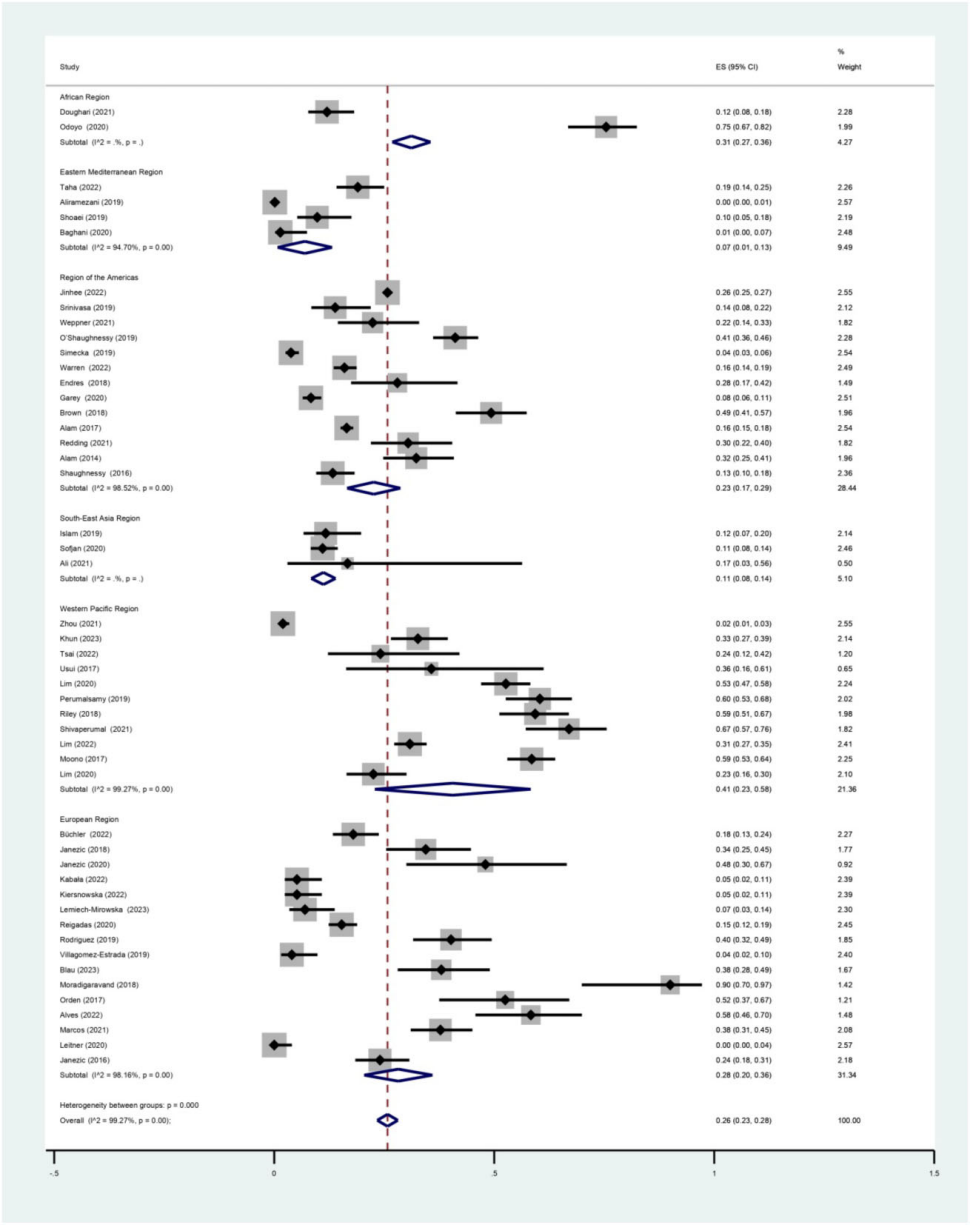
Forest plot of the pooled prevalence of *C. difficile* contamination in healthcare and non-healthcare environments according to region.

The pooled prevalence of *C. difficile* contamination according to region indicated that the highest prevalence was in the Western Pacific with a prevalence of 0.41 (95% CI 0.23 to 0.58). The lowest prevalence was in the Eastern Mediterranean with a prevalence of 0.07 (95% CI 0.01 to 0.13). The pooled prevalence in the other regions was 0.40, 0.31, 0.23 and 0.11 in Europe, Africa, America and South-East Asia, respectively. Complete results by region are shown in Table 2 and Figure 4.

The pooled prevalence of *C. difficile* contamination according to setting was 0.18 (95% CI 0.16 to 0.20) in the healthcare environment and 0.36 (95% CI 0.30 to 0.42) in the non-healthcare environment (Figure 3).

The pooled prevalence of *C. difficile* contamination according to sample type was 0.59 (95% CI 0.47 to 0.71) in soil samples and 0.34 in shoe samples (95% CI 0.32 to 0.45).

### Prevalence of *C. difficile* contamination in healthcare environments

In total, among 11 475 environmental samples in healthcare settings, 1962 were positive for *C. difficile* contamination. The pooled prevalence of *C. difficile* contamination among environmental samples in healthcare settings was 0.18 (95% CI 0.16 to 0.20).

The pooled prevalence of *C. difficile* contamination in healthcare environments according to region indicated that the highest prevalence was found in the Western Pacific (0.33; 95% CI 0.10 to 0.56), followed by Africa (0.31; 95% CI 0.27 to 0.36). The prevalence for all regions is shown in Table 3.

**Table 3.** Pooled prevalence of *C. difficile* contamination in healthcare and non-healthcare environments

						Heterogeneity			
Independent variable	Number of studies	Sample size	Total number positive	Pooled prevalence	95% CI	Q statistic	I^2^ (%)	p	Heterogeneity between subgroup bias
**Healthcare**									
Region									
Africa	2	264	104	0.31	0.27–0.36	-	-	-	0.00
Eastern Mediterranean	2	1527	11	0.00	0.00–0.00	-	-	-	
America	9	6359	1193	0.19	0.12–0.26	449.47	98.22	0.00	
South-East Asia	1	400	44	0.11	0.08–0.14	-	-	-	
Western Pacific	5	1819	481	0.33	0.10–0.56	695.26	99.42	0.00	
Europe	6	1106	129	0.08	0.02–0.15	149.67	96.66	0.00	
Sample type									
Bed	7	447	43	0.09	0.04–0.15	53.52	88.79	0.00	0.00
Chair	2	246	16	0.03	0.01–0.05	-	-	-	
Computer	3	108	7	0.04	0.00–0.09	-	-	-	
Door handle	2	32	1	0.00	0.00–0.01	-	-	-	
Light switch	2	98	0	0.00	0.00–0.00	-	-	-	
Shoe	3	643	205	0.34	0.17–0.51	-	-	-	
Soil	2	189	121	0.66	0.59–0.73	-	-	-	
Table	3	76	9	0.10	0.03–0.17	-	-	-	
Toilet or bathroom	5	413	97	0.14	0.01–0.28	98.88	95.95	0.00	
Wheelchair	2	40	5	0.12	0.02–0.22	-	-	-	
Window	2	37	4	0.01	0.00–0.04	-	-	-	
**Non-healthcare**									
Location									
Public	16	6797	1869	0.38	0.28–0.48	705.04	98.01	0.00	0.00
Home	6	1607	345	0.33	0.20–0.45	138.87	96.32	0.00	
Farm	7	800	327	0.42	0.31–0.53	54.79	89.05	0.00	
Veterinary hospital	1	100	4	0.04	0.02–0.10	-	-	-	

The pooled prevalence of *C. difficile* contamination according to sample type indicated that the highest contamination rates were in soil and shoes, with a prevalence of 0.66 (95% CI 0.59 to 0.73) and 0.34 (95% CI 0.17 to 0.51), respectively. This was followed by toilet or bathroom with a prevalence of 0.14 (95% CI 0.01 to 0.28), then wheelchair with a prevalence of 0.12 (95% CI 0.02 to 0.22). Complete results due to the prevalence of various sample types in healthcare settings are shown in Table 3.

### Prevalence of *C. difficile* contamination in non-healthcare environments

Overall, among 9304 non-healthcare environmental samples, 2545 were positive for *C. difficile* contamination. The pooled prevalence was 0.36 (95% CI 0.30 to 0.41). The prevalence in the non-healthcare environment according to location was 0.42, 0.38, 0.33 and 0.04 on farms, in public places, in the home and veterinary hospital, respectively.

## Discussion

*Clostridioides difficile* is a Gram-positive, spore-forming anaerobic bacterium that is the cause of antibiotic-associated diarrhea, toxic megacolon and pseudomembranous colitis, which may be life-threatening.^58^ The bacterium is resistant to a wide range of bactericide substances due to the endospore, which can survive for months in harsh environments. This study is the first global comprehensive systematic review concerning *C. difficile* contamination in healthcare and non-healthcare environments and summarizes its prevalence over the last decade (2014–2024).

In the current study, the pooled prevalence of *C. difficile* contamination in all environmental samples was estimated as 0.26, but was different for various levels of other variables such as diagnostic test and setting. This result indicates the notable presence of *C. difficile* in diverse environments across the world in the last decade. On the other hand, research data suggest that *C. difficile* can persist on surfaces for months. Also, the bacterium has shown resistance to various chemical agents and disinfectants in recent years. Therefore, to effectively control CDI rates, it is crucial to implement a standardized surveillance program with attention paid to the role of the environment in the transmission of the disease. In recent years, more attention and effort have been dedicated to controlling CDI, especially healthcare-associated CDI, and some countries have established guidelines for the detection and control of infection. However, there is a need to implement these programs and guidelines on a global scale, taking account of the role of the environment in the spread of the disease.

Based on our results, the pooled prevalence was 0.31 (95% CI 0.25 to 0.38) and 0.24 (95% CI 0.19 to 0.30) before and after 2020, respectively, which indirectly shows the decrease in CDI cases in recent years. Research indicates that patients treated with certain antibiotics are less likely to contaminate their surroundings, demonstrating a direct link between infection management and environmental hygiene.^59^ Therefore, the decreased contamination rate in the environment in recent years can be attributed to implementing a control program of CDI in several countries, especially in the healthcare system. For example, there is increasingly promising evidence that CDI rates have declined in the healthcare systems of Europe and the USA that should be implemented in other countries.

Screening is an important method for disease control, for reducing symptoms and costs. Choosing screening-accurate tools to identify contaminated samples should be considered. Assessments of diagnostic testing, extracted from studies, were included in this study. Our results based on the diagnostic method utilized indicated that the pooled prevalence was an estimated 26% (95% CI 21 to 32%) using a culture test, approximately equal to those found with a culture test plus another method, attributable to other tests being used less than culture in a large number of studies, although it was an estimated 62% (95% CI 50 to 74%) for PCR only. Most studies found that the molecular-based method is more sensitive and more specific than the culture test; therefore, it appears that PCR is suitable for screening.

Our findings indicated a pooled prevalence of 18% within the healthcare system, whereas the pooled prevalence for the non-healthcare system was 36%. However, we cannot definitively conclude that the prevalence is lower in healthcare compared with non-healthcare settings. This is because a significant portion of the non-healthcare samples were associated with public places, such as those from wastewater treatment plants and agricultural sources, including animal feces, manure and compost, which influenced our results. In any case, environmental contamination in healthcare plays an important role in the dissemination of *C. difficile* infection between hospital staff and patients, leading to an increase in hospital-acquired cases.

Our results for the healthcare environment indicate that shoes, toilet or bathroom and wheelchairs have the highest contamination rates. Shoe soles of healthcare personnel and community-dwelling individuals can become colonized by microorganisms through direct or indirect transmission routes. Thus, shoe soles can be recognized as agents for the spread of *C. difficile* spores. Exposure of shoe soles to the ultraviolet-C (UVC) device significantly decreased the contamination rate of bacteria. It is recommended that UVC devices are utilized in healthcare environments to lower the number of colony-forming units of bacteria on shoe soles, which will consequently reduce the colonization of other surfaces.

The results showed that the non-healthcare environment has a remarkable contamination rate for *C. difficile*. The contamination rate on farms and in the public environment was slightly higher than in the home environment. This can be attributed to the fact that, in the home environment, surfaces are continually being disinfected. It is important to note that the overall contamination rate in home environments could be significantly lower than reported, because most of the home samples were taken from shoes, soil and toilets.

Among non-healthcare environments, farms have the highest prevalence. The bacterium has been isolated from various surfaces in farms, including soil, sand and mud. It was suggested that contaminated animals could be a reservoir of *C. difficile* for transmission to humans.^58^ The genetic similarity of *C. difficile* strains found in animals and humans reinforces the idea that animal farms can serve as reservoirs for human infections.^60,61^ On the other hand, studies have shown that animals, especially pigs, cattle and poultry, can shed the bacterium in their feces, contributing to its presence in the farm environment.^58^ It seems that the transmission chain between animals and the community has a role in the increase of community-acquired cases. This result highlights the importance of monitoring and controlling the presence of *C. difficile* in agricultural settings to mitigate the risk of infection in the human population.

In addition to farms, our results indicates the presence of the bacterium in various public places, such as wastewater treatment plants, where it was detected at various stages of the treatment process. This indicates that the bacterium can endure the treatment processes typically employed at these facilities, raising concerns about its potential release into the environment and subsequent risk to public health. The presence of *C. difficile* in wastewater suggests that these environments could serve as reservoirs for community-associated CDI. Hence, wastewater environments are critical in the transmission dynamics of *C. difficile*, necessitating enhanced monitoring and treatment strategies to mitigate the risks associated with this pathogen in public health contexts.

Overall, we report a notable global contamination rate for *C. difficile* among diverse environments over the last decade. Based on evidence, the risk of acquired environmental infection with *C. difficile* was similar to the risk of contact with an infected person.^62^ Therefore, environmental contamination with *C. difficile* plays an important role in increasing the number of reservoirs of *C. difficile* spores, leading to an increase in community-acquired cases. Therefore, there is a need to implement programs and guidelines on a global scale, taking account of the role of the environment in the spread of the disease.

The strengths of this study include its large total sample size and extraction of detailed data. However, this study has some limitations, and the results presented here should be interpreted with regard to these limitations. One primary limitation of this review is the possibility that our search strategy missed some studies. To address this limitation, we utilized PubMed and Google Scholar, both of which cover a wide range of scientific journals. Additionally, we conducted a thorough search of the references cited in eligible papers to identify any further relevant studies. Another limitation concerns the uneven distribution of studies across different strata, such as region. After stratifying the results by various variables, some strata had relatively few studies, which may have impacted the reliability of the prevalence estimates for those strata. We have transparently described the characteristics and number of studies per strata in the current article to ensure readers are aware of this limitation. Finally, it is important to highlight that the type of environmental sample is the key variable likely affecting prevalence comparisons across different strata. Nonetheless, we have tried to interpret the results with this factor in mind to offer a more nuanced understanding of the findings.

## Conclusions

The findings of our study over the past decade reveal a significant prevalence of *C. difficile* contamination across various environments worldwide. This alarming trend underscores a pressing need to prioritize environmental factors and implement effective disinfection strategies as part of comprehensive prevention and control programs aimed at tackling this challenging disease. By addressing the environmental reservoirs of *C. difficile*, we can take proactive steps to mitigate the spread of this pathogen and improve public health outcomes.

## Data Availability

We are committed to sharing the data upon rational request.
